# RCBTB1 Deletion Is Associated with Metastatic Outcome and Contributes to Docetaxel Resistance in Nontranslocation-Related Pleomorphic Sarcomas

**DOI:** 10.3390/cancers11010081

**Published:** 2019-01-11

**Authors:** Olivier Mauduit, Céline Brulard, Tom Lesluyes, Vanessa Delcroix, Gaëlle Pérot, Nina Choublier, Mickael Michaud, Jessica Baud, Pauline Lagarde, Alain Aurias, Jean-Michel Coindre, Lydia Lartigue, Jean-Yves Blay, Frédéric Chibon

**Affiliations:** 1 Inserm U1218, Bergonié Cancer Institute, F-33076 Bordeaux, France; olivier.mauduit@live.fr (O.M.); celine.brulard@univ-tours.fr (C.B.); tom.lesluyes@inserm.fr (T.L.); vanessa.delcroix@orange.fr (V.D.); gaelle.perot@inserm.fr (G.P.); nina.choublier@inserm.fr (N.C.); mickael_michaud@yahoo.fr (M.M.); j.massiere@bordeaux.unicancer.fr (J.B.); pauline.lagarde@wanadoo.fr (P.L.); alain.aurias@hotmail.fr (A.A.); J.Coindre@bordeaux.unicancer.fr (J.-M.C.); lydia.lartigue@gmail.com (L.L.); 2 ED 340 BMIC, Claude Bernard Lyon 1 University, F-69622 Villeurbanne, France; 3 Department of Pathology, Bergonié Cancer Institute, F-33076 Bordeaux, France; 4 Department of Life and Health Sciences, University of Bordeaux, F-33000 Bordeaux, France; 5 Department of Life Sciences, University of Orléans, F-45100 Orléans, France; 6 Department of Pathology, Léon Bérard Center, F-69003 Lyon, France; jean-yves.blay@lyon.unicancer.fr; 7 INSERM U1037, Cancer Research Center of Toulouse (CRCT) and Department of Pathology, Institut Claudius Regaud, IUCT-Oncopole, 31037 Toulouse, France

**Keywords:** sarcoma with complex genomics, pleomorphic sarcoma, RCBTB1, docetaxel, cancer

## Abstract

Half of soft-tissue sarcomas are tumors with complex genomics, which display no specific genetic alterations and respond poorly to treatment. It is therefore necessary to find new therapeutic targets for these sarcomas. Despite genetic heterogeneity across samples, oncogenesis may be driven by common pathway alterations. Therefore, genomic and transcriptomic profiles of 106 sarcomas with complex genomics were analyzed to identify common pathways with altered genes. This brought out a gene belonging to the “cell cycle” biological pathway, *RCBTB1* (RCC1 And BTB Domain Containing Protein 1), which is lost and downregulated in 62.5% of metastatic tumors against 34% of non-metastatic tumors. A retrospective study of three sarcoma cohorts revealed that low *RCBTB1* expression is prognostic for metastatic progression, specifically in patients that received chemotherapy. In vitro and in vivo, RCBTB1 overexpression in leiomyosarcoma cells specifically sensitized to docetaxel-induced apoptosis. This was associated with increased mitotic rate in vitro and higher growth rate of xenografts. By contrast, *RCBTB1* inhibition decreased cell proliferation and protected sarcoma cells from apoptosis induced by docetaxel. Collectively, these data evidenced that *RCBTB1* is frequently deleted in sarcomas with complex genomics and that its downregulation is associated with a higher risk of developing metastasis for patients receiving chemotherapy, likely due to their higher resistance to docetaxel.

## 1. Introduction

Sarcomas are a rare group of malignant tumors that arise from mesenchymal tissue. Molecular approaches have described three main genetics in these tumors: reciprocal translocations, specific mutations, and complex genomic profiles [1]. Soft tissue sarcomas (STSs) with complex genomics, which represent 50% of STSs, display no specific genetic alterations [2]. These tumors form a heterogeneous group with numerous histotypes such as leiomyosarcomas (LMSs), undifferentiated pleomorphic sarcomas (UPSs), myxofibrosarcomas, pleomorphic liposarcomas, and pleomorphic rhabdomyosarcomas. Patient outcome is mainly driven by the intrinsic tumor biology and aggressiveness but also by tumor response to systemic chemotherapy. The response rate to chemotherapy ranges from 20% to 60% during first-line chemotherapy with doxorubicin, the standard first-line treatment for the last 40 years [2]. For metastatic sarcomas, the combination of gemcitabine and docetaxel provides a median overall survival (OS) of 18 months [3]. Other molecules such as pazopanib [3], regorafenib [4], and trabectidine [5] have also shown efficacy after failure of standard chemotherapy. More recently, a novel monoclonal antibody against platelet-derived growth factor receptor alpha (olaratumab) has been proposed as a first-line treatment in combination with doxorubicin. This drug is currently under phase III clinical evaluation [6]. Since surgery is the current best treatment available against these neoplasms, it is necessary to enhance the efficiency of chemotherapy by improving our understanding of the mechanisms involved in drug resistance or by finding new therapeutic targets and biomarkers of tumor response.

These tumors are characterized by a complex karyotype. At the genomic level, there are numerous gains and losses of chromosomes or chromosome regions, mostly variable across tumors [1]. Some recurring genetic alterations have been identified in these tumors, i.e., losses of chromosomes 10, 13, and 16 [7]. Among them, the most frequent is the 13q14-21 region loss in LMSs and UPSs, leading to RB1 deletion or inactivation [8]. However, no driver gene specific to the oncogenesis of these tumors has been identified so far.

In order to identify new genes involved in sarcoma oncogenesis, we applied an approach integrating clinical, genomic and transcriptomic data from 106 sarcomas samples [9]. Since sarcomas with complex genetic profiles have many alterations, it is challenging to distinguish drivers from passengers. We hypothesized that some altered biological pathways are common to all sarcomas or specific to a sub-group (such as histotype, localization, prognosis, and clinical evolution). By selecting altered genes belonging to biological pathways dysregulated in all sarcomas, we identified *RCBTB1* (RCC1 And BTB Domain Containing Protein 1), for which downregulation is associated with metastatic progression.

## 2. Results

### 2.1. Identification of a Gene Associated with Metastatic Progression

To discriminate driver and passenger alterations, we hypothesized that oncogenesis and tumoral progression are driven by recurrent pathway alterations shared by sarcomas with complex genomics. In order to identify such pathways, array comparative genomic hybridization (aCGH) profiles of 106 sarcomas (Table 1) were first analyzed. We identified an average of 2960 altered genes (with tumor-specific copy number variations, CNVs) per sample, with similar proportions of gains and losses in tumors: 53% and 47%, respectively. This represents a total of 12,124 genes altered in this cohort. In order to identify driver genes, only the 8527 genes whose expression (determined by cDNA microarray) was significantly modified according to their genomic alteration (upregulation for gains and downregulation for losses) were retained for further analysis.

Using this gene list and the Gene Ontology (GO) database [10], we obtained a total of 7446 altered GO biological pathways. We then selected the 41 biological pathways (Supplementary Table S1) that were altered in each of the 106 tumors. They consisted of three main groups: cellular, metabolic and developmental processes, which are involved in various cellular functions: signaling, cell organization, acid nucleic metabolism, differentiation, and cell cycle (Supplementary Table S1).

**Figure 1:**
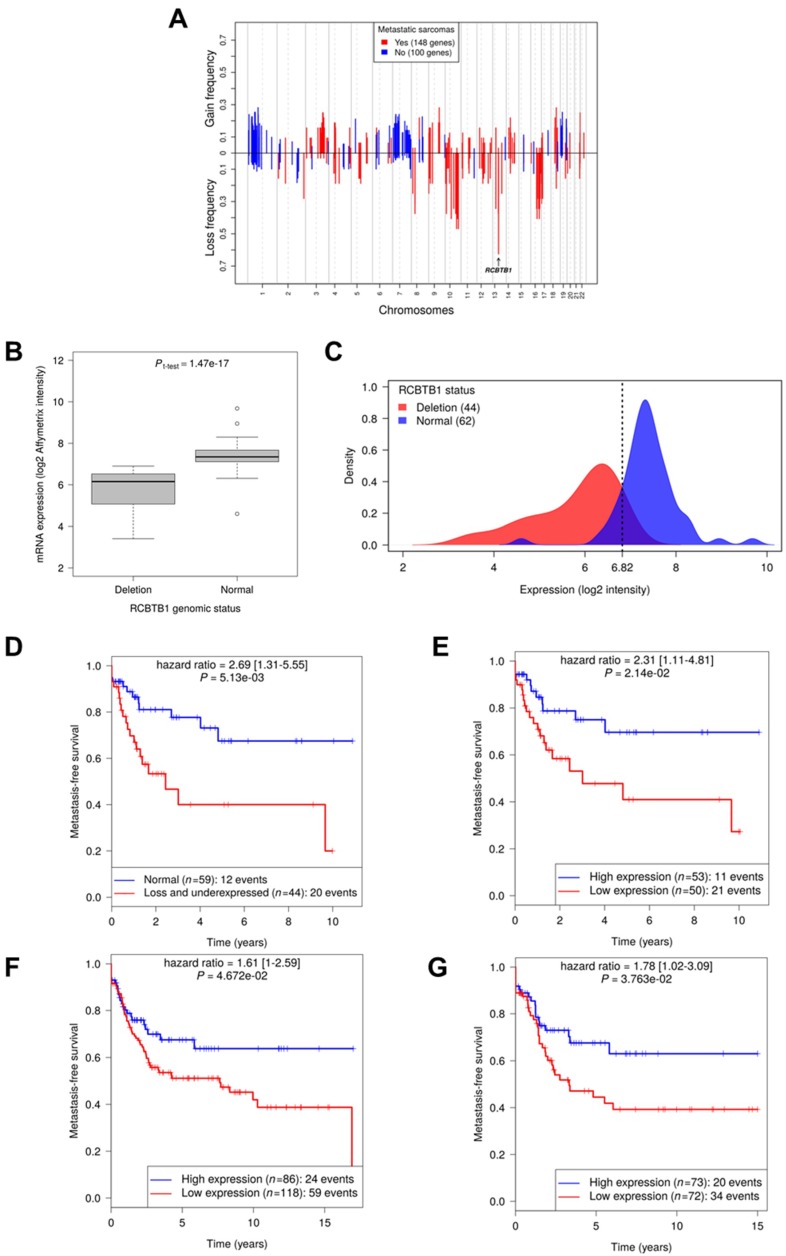
Association of *RCBTB1* deletion with metastatic evolution. (**A**) Each bar represents a gene on its chromosomal location and its alteration frequency: positive for a gain or negative for a deletion. In red: 151 altered genes are significantly correlated with metastatic evolution. In blue: 100 other altered genes are significantly associated with no metastatic evolution. (**B**,**C**) Analysis of *RCBTB1* expression according to its genomic status (normal or deleted), represented (**B**) on a boxplot showing that *RCBTB1* deletion is significantly associated with a decrease of its expression (*p* = 1.47e-17) and (**C**) on a density curve comparing signal intensities between the population of tumors with a normal copy number of *RCBTB1* and tumors harboring *RCBTB1* deletion. The threshold = 6.82 obtained defines low expression for (**D**,**E**) metastasis-free survival (MFS) in patient cohort #1, split according to (**D**) *RCBTB1* genomic status (normal or deleted and under-expressed) or (**E**) expression level of *RCBTB1* determined by microarrays. The prognostic value of *RCBTB1* expression (cut-off = mean) on metastasis-free evolution was also investigated in cohorts #2 (**F**) and #3 (**G**).

All the selected genes involved in these 41 biological pathways were analyzed in light of metastatic evolution by identifying genes that are significantly enriched in metastatic tumors compared to non-metastatic tumors. This selection step filtered out 248 altered genes significantly associated with metastatic evolution including *RCBTB1*, a gene belonging to the cell cycle biological pathway (GO:0007049). *RCBTB1* is the most frequently deleted gene significantly associated with metastatic evolution (Chi-squared test *p* = 6.44e-3, lost in 63% of metastatic tumors against 34% of non-metastatic tumors (Figure 1A). In addition, the most frequently deleted histotype is in LMSs, with 58% RCBTB1 deletion against 36% in the rest of the cohort #1 (UPSs, myxofibrosarcomas, pleomorphic liposarcomas, dedifferentiated liposarcomas, and other sarcomas combined) (Chi-squared test; *p* = 3.64e-2).

Next, we tested whether *RCBTB1* may be a prognostic factor for tumor progression. *RCBTB1* expression is significantly correlated to its matching genomic status (Figure 1B). The intersection between the curves showing the distribution of *RCBTB1* expression according to its genomic status (deleted versus normal) (Figure 1C) defines the value of 6.82 that is the threshold between low and high expression of *RCBTB1*. *RCBTB1* deletion and low expression correlate with a significant higher risk for developing metastasis (Figure 1D,E). The prognostic value of *RCBTB1* expression level was further validated in a second independent cohort of 204 sarcoma patients (cohort #2 described in Table 1) (Figure 1F) and in 145 sarcomas for which *RCBTB1* expression was determined by RNA sequencing (cohort #3 [11]) (Figure 1G). Thus, in three cohorts, lower *RCBTB1* expression is significantly associated with an increased risk of developing metastasis.

With *RCBTB1* likely acting as a tumor suppressor gene, we searched for second event mutations that could occur in addition to deletions. In cohort #3 (analyzed by RNA sequencing), 12 candidate variants were reported in exonic regions. Eleven variants were synonymous and one was a missense variant (NM_018191:p.A24V) already known as a common single nucleotide polymorphism, described in the dbSNP database (rs4942848) and in the 1000 G project (allele frequency: 0.59). We thus concluded that *RCBTB1* seems to be mainly downregulated by deletion mechanisms.

These results were validated by analyzing the TCGA data available on cBioPortal database [12,13]. Among the 261 patients, we selected only sarcoma samples for which we had all of the information about *RCBTB1* genomic status and mRNA expression, histotype, and metastatic status. One missense mutation was reported (H325N) but was annotated as benign by the PolyPhen-2 web tool. We note that, among the 162 tumors, 13% and 61% harbor a homozygous and a heterozygous deletion of *RCBTB1*, respectively (Supplementary Table S2). The most frequently deleted histotypes are LMS and UPS. In this cohort, *RCBTB1* expression is also significantly reduced in deleted samples compared to non-deleted tumors (Supplementary Figure S1). Moreover, low *RCBTB1* expression is significantly associated with metastatic disease (Chi-squared test; *p* = 0.046) (Supplementary Table S3).

Given that *RCBTB1* has been reported to be involved in drug resistance [14,15], we investigated a potential impact of *RCBTB1* expression level on the chemosensitivity of sarcomas with complex genomics.

### 2.2. Impact of RCBTB1 on Drug Response

**Figure 2:**
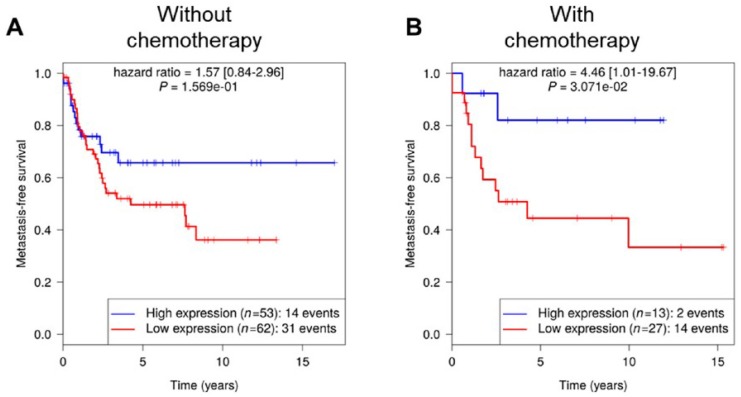
*RCBTB1* is significantly associated with metastasis-free survival for patients who received chemotherapy. Metastasis-free survival (MFS) for sarcoma patients is split according to the *RCBTB1* expression level determined by microarrays. Cohorts correspond to patients that did not (**A**) or did (**B**) receive chemotherapy.

In cohort #2, for which treatment conditions were available, *RCBTB1* prognostic value is restricted to patients receiving chemotherapy (Figure 2). Unfortunately, detailed chemotherapy regimens are not available in this cohort. To circumvent this lack of information, current drugs for pleomorphic sarcoma treatment [2] (doxorubicin, gemcitabine, and docetaxel) were screened and apoptotic responses of LMS cell lines (the main histotype affected by *RCBTB1* deletions) with induced *RCBTB1* expression were measured.

**Figure 3:**
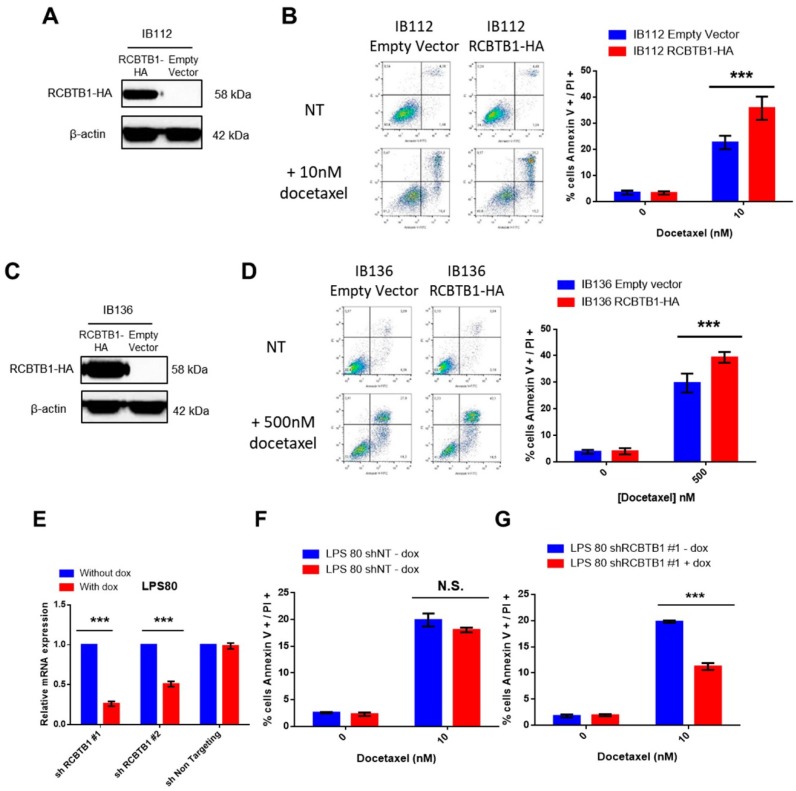
RCBTB1 expression level modulates in vitro the sensitivity of sarcoma cell lines to docetaxel. (**A**–**D**) Validation of RCBTB1-overexpressing cells after lentiviral transduction and assessment of cell death induced by docetaxel in (**A**,**B**) IB112 and (**C**,**D**) IB136 cell lines. (**A**,**C**) Validation of RCBTB1-HA expression by western blotting after lentiviral transduction in (**A**) IB112 and (**C**) IB136 cell lines. Control cell lines express an empty vector. The signal detected corresponds to the HA-tag fused to RCBTB1. (**B**,**D**) Apoptosis induced by 72 h of treatment with docetaxel in (**B**) IB112 and (**D**) IB136 cells was measured by dual Annexin V-FITC/PI staining and analyzed by flow cytometry. Quadrants are representative of the results obtained for all experiments. Histograms summarize the results of three independent experiments with three replicates. Data are represented as mean (SD). (**E**) Validation of *RCBTB1* downregulation by Taqman experiments after lentiviral transduction of shRNAs targeting *RCBTB1* in LPS80 cell line. Control cell line expresses a shRNA targeting no human sequence. Expression of shRNAs was induced by addition of doxycycline into culture medium. Histogram summarizes three independent experiments. (**F**,**G**) Cells were treated with or without doxycycline to induce expression of (**F**) non-targeting shRNA or (**G**) shRCBTB1#1 over 3 days. Then, apoptosis induced by 48 h of treatment with docetaxel was measured by dual Annexin V-FITC/PI staining by flow cytometry. Data shown are representative of three independent experiments with three replicates. *** *p* < 0.001, N.S.: not significant

The first LMS cell line (IB112) has a homozygous deletion of *RCBTB1* and so, has no endogenous *RCBTB1* expression. After lentiviral transduction and validation of *RCBTB1* expression (HA)-tagged, because none of the tested antibodies was specific to RCBTB1) by Western blotting (Figure 3A), cell death was assessed after incubation with chemotherapies. Although no significant difference was observed with doxorubicin or gemcitabine (Supplementary Figure S2), the apoptosis rate was significantly higher in RCBTB1-expressing cells compared to control cell line (IB112 empty vector) after 72 h of incubation with 10 nM docetaxel. Indeed, in IB112-RCBTB1-HA cells, dual Annexin V- fluorescein isothiocyanate/propidium iodide (FITC/PI) staining occurs in 35.8% of cells, whereas control cell line shows 22.6% of cell death after docetaxel treatment (Figure 3B).

The same experiments were repeated in another LMS cell line (IB136) that bears a heterozygous deletion of *RCBTB1* and is more resistant to docetaxel than the IB112 cell line (500 nM of docetaxel was used during 72 h to induce an apoptotic rate comparable to IB112 cells). Likewise, *RCBTB1* overexpression in IB136 cells (Figure 3C) increases average cell death from 29.7% in control cell line to 39.4% after incubation with docetaxel (Figure 3D).

To confirm that *RCBTB1* expression level could impact response to docetaxel, we analyzed the effect of RCBTB1 downregulation in a dedifferentiated liposarcoma cell line (LPS80), which is not deleted for *RCBTB1* and expresses the most RCBTB1 among our sarcoma cell lines, according to Taqman experiments. LPS80 cells were transduced with two different inducible shRNA targeting *RCBTB1* and one shRNA non-targeting (shNT) *RCBTB1* as a negative control. First, inhibition of *RCBTB1* was validated by Taqman experiment. When shRCBTB1 #1 and #2 expression was induced by doxycycline, we observed a significant inhibition of *RCBTB1* mRNA of 74% and 49%, respectively (Figure 3E). Then, we evaluated the effect of shRNA#1 (that mostly inhibits *RCBTB1* expression) on apoptosis induced by docetaxel. No significant difference was observed when shNT is induced as compared to the condition without doxycycline (Figure 3F). By contrast, downregulation of RCBTB1 by shRNA#1 significantly reduced cell death induced by docetaxel, as demonstrated by dual Annexin V-FITC/PI staining reaching 19.8% in control cells whereas cell death rate occurs only in 11.2% of cells cultured with doxycycline (Figure 3G).

Considering that *RCBTB1* downregulation is associated with metastatic development for patients receiving chemotherapy, we also assessed the migratory capacities of the homozygous deleted IB112 cell line. Wound-healing assays did not evidence any significant impact of *RCBTB1* overexpression on cell migration (Supplementary Figure S3), thereby suggesting that the prognostic value of *RCBTB1* mainly relies on its effect on tumor response to treatment.

### 2.3. Impact of RCBTB1 Overexpression In Vivo

Then, to confirm our previous observations on drug response, antitumor efficacy of docetaxel depending on *RCBTB1* expression level was tested on sarcoma xenografts. Only the IB136 cell line was able to generate subcutaneous tumors after implantation into mice.

**Figure 4:**
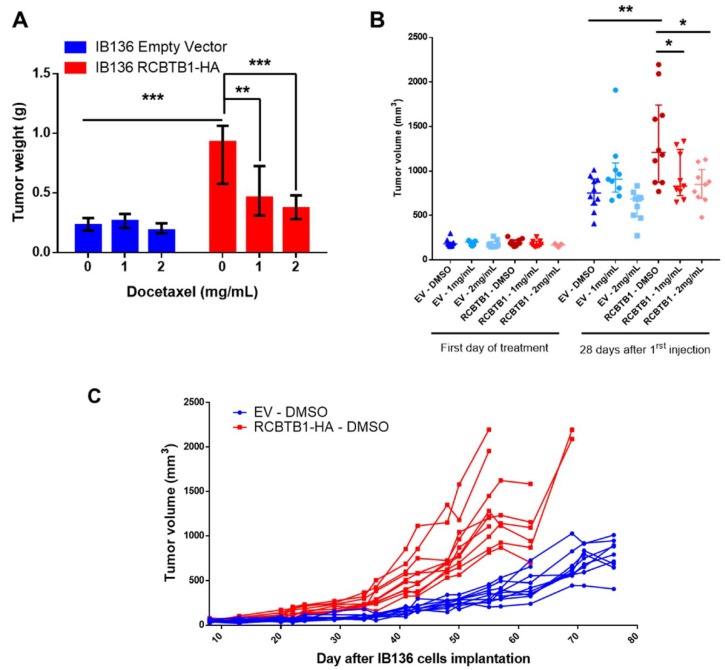
RCBTB1 overexpression increases tumor sensitivity to docetaxel, but also promotes tumor growth in mice. (**A**) Tumor weight after 28 days treatment (0: DMSO; 1: 1 mg/mL of docetaxel and 2: 2 mg/mL of docetaxel). Each group was composed of 10 mice. Histograms sum up results with median and interquartile range (IQR). (**B**) Tumor volume at the beginning and at the end of treatment with DMSO or docetaxel (1 mg/mL and 2 mg/mL) in each group (EV = tumors produced by IB136 empty vector cells; RCBTB1 = tumors formed by IB136 RCBTB1-HA cells). Line in the middle is the median, errors bars represent IQR. Individual points represent the volume of one tumor. (**C**) Follow-up of individual tumor volume in groups treated with DMSO since the day of cell implantation (IB136 empty vector cells in blue, IB136 RCBTB1-HA in red) until sacrifice. * *p* < 0.05, ** *p* < 0.01, *** *p* < 0.001.

In accordance with in vitro results, mice bearing IB136 RCBTB1-HA tumors harbored a significant reduction of tumor weight after treatments, with on average a 49.3% and a 56.7% reduction after 1 mg/mL and 2 mg/mL of docetaxel, respectively (Figure 4A). By contrast, in mice engrafted with IB136 empty vector (EV) cells, docetaxel had no significant impact on tumor weight. Similar observations were made by comparing tumor volumes at the end of the treatment, with an average volume of 1353 mm^3^ for IB136 RCBTB1-HA tumors in the placebo group as opposed to 936 and 835 mm^3^ in the groups treated with 1 mg/mL and 2 mg/mL of docetaxel, respectively (Figure 4B). Follow-up of tumor volume from the first injection of treatments shows that docetaxel significantly reduces tumor growth from 1 mg/mL only in mice engrafted with IB136 RCBTB1-HA cells (Supplemental Figure S4A,B).

Nevertheless, a surprising result arose from these in vivo experiments. Indeed, tumor volume was significantly higher for IB136 RCBTB1-HA tumors than IB136-EV, when both were treated with DMSO (Figure 4B), from day 9 post-treatment (Supplementary Figure S4C). This was confirmed by weighing tumors at the end of DMSO treatment, with an average of 260 mg and 890 mg for tumors produced by IB136-EV and IB136 RCBTB1-HA cell lines, respectively (Figure 4A). Follow-up of individual tumor volumes from the day of cell implantation clearly indicates that tumor growth is delayed in mice engrafted with IB136 empty vector cells compared to those bearing IB136 RCBTB1-HA tumors (Figure 4C).

In light of this unexpected effect of RCBTB1 on tumor growth and considering that docetaxel specifically inhibits microtubule depolymerization during mitosis, we next investigated whether *RCBTB1* expression level modulates mitotic rate in sarcoma cells.

### 2.4. Impact of RCBTB1 Expression on Mitosis

To understand how *RCBTB1*-expressing cells are more sensitive to docetaxel, we analyzed the proportion of mitotic LMS cells with or without docetaxel treatment.

**Figure 5:**
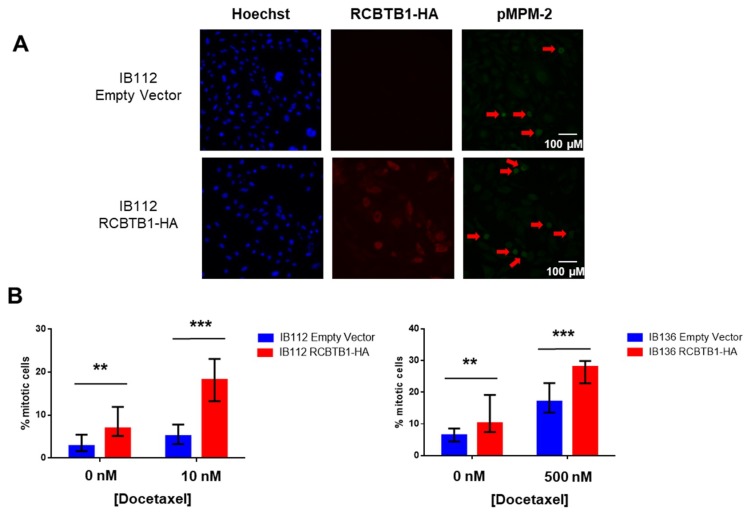

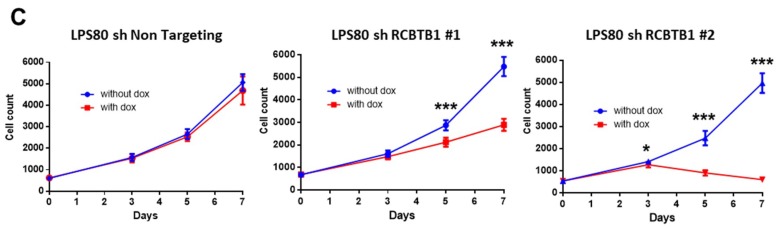
RCBTB1 overexpression increases the percentage of mitotic cells in two leiomyosarcoma (LMS) cell lines, whereas its downregulation reduces cell proliferation in a dedifferentiated liposarcoma cell line. (**A**,**B**) RCBTB1 was overexpressed by lentiviral transduction in IB112 and IB136. Cells were treated with docetaxel (at 10 nM and 500 nM for IB112 and IB136, respectively) for 24 h and then were fixed for fluorescent staining. Nuclei were stained with Hoechst. Mitosis-specific marker pMPM-2 (green) and RCBTB1-HA (red) dual staining was analyzed by epifluorescence microscopy. (**A**) These images are representative of the results obtained with IB112 LMS cell lines Scale bare: 100 µM. (**B**) Histograms sum up results that are representative of three independent experiments. Data shown correspond to median (IQR). (**C**) Cell proliferation of the LPS80 cell line expressing the different shRNAs was measured by cell counting using a flow cytometer and compared to respective conditions without doxycycline. Data shown are representative of three independent experiments with eight replicates and represented as mean (SD). * *p* < 0.05, ** *p* < 0.01, *** *p* < 0.001.

To that purpose, LMS cell lines were stained with an antibody targeting phosphorylated MPM2 (Figure 5A,B), a well-known marker of mitosis [16]. In both LMS cell lines, RCBTB1 (labeled with anti-HA-tag antibody) expression is associated with a significantly higher percentage of mitotic cells compared to respective control cell lines, in control conditions and after docetaxel treatment (Figure 5B). Indeed, in control conditions, we observed on average 4% and 9% of mitotic cells in IB112 empty vector and IB112-RCBTB1-HA cells, respectively. Regarding IB136 cell lines, mitotic cells account for 7% and 14% of control and RCBTB1-overexpressing cells, respectively. After 24 h incubation with docetaxel, mitotic cells represent 5.4% and 18% in IB112 and IB136 control cell lines, respectively, vs. 19.1% and 27.2% in RCBTB1 overexpressing in IB112 and IB136 cell lines, respectively.

RCBTB1 expression appears to stimulate mitosis and, in turn, proliferation, thus making cells more sensitive to docetaxel treatment. In line with this observation, inhibition of RCBTB1 in LPS80 cell line dramatically decreased the number of cells among days: from 5479 to 2893 cells with shRNA#1 and from 4567 to 600 cells with shRNA#2 on average after 7 days (Figure 5C). On the contrary, shNT induction did not significantly affect cell proliferation.

Altogether, these results indicate that the sensitization effect of *RCBTB1* expression on docetaxel-induced apoptosis is at least due to a higher proportion of mitotic cells, which results in greater tumor growth in vivo as observed with IB136 cell line. Reciprocally, increased resistance to docetaxel of LPS80 expressing shRCBTB1 is associated with reduced cell proliferation.

## 3. Discussion

Sarcomas with complex genomics are rare and aggressive tumors, characterized by a high risk of metastasis, and consequently a poor overall survival. To improve patient care, it is important to understand their oncogenesis.

For that purpose, we first identified all the quantitatively altered genes and retained only those that are part of the biological pathways altered in 100% of sarcomas with complex genomics. This bioinformatic approach, based on commonly altered biological pathways instead of genes, allowed us to identify a gene significantly associated with metastatic progression. Here, we report that among the several genes located within the frequently deleted 13q14 band [8], RCBTB1 was filtered out for its impact on the cell cycle pathway. aCGH and RNA sequencing revealed that this gene is mainly inactivated by a deletion mechanism. RCBTB1 downregulation is associated with metastatic evolution in sarcomas with complex genetics. Interestingly, the prognostic value of RCBTB1 was restricted to patients receiving chemotherapy. Altogether, these observations led us to formulate the hypothesis that RCBTB1 may be a tumor suppressor gene whose activity could be related to drug response.

Data reported here evidenced that *RCBTB1* expression impacts docetaxel-induced apoptosis in dedifferentiated liposarcoma (LPS80) and LMS (IB112, IB136) cell lines. In LPS80 cells, *RCBTB1* inhibition conferred resistance to docetaxel. Consistent with this observation, Lee et al. (2013) reported that *RCBTB1* inhibition protects in vitro liposarcoma cells from apoptosis induced by nocodazole [14], another inhibitor of microtubule polymerization. Conversely, in LMS cell lines overexpressing RCBTB1, we observed an enhanced apoptosis after docetaxel treatment. In vivo, IB136-RCBTB1-HA tumors were more sensitive to docetaxel treatment than tumors arising from the control cell line. These mechanistic insights should explain why the prognostic value of RCBTB1 is restricted to patients receiving chemotherapy. RCBTB1 expression loss or downregulation may represent a selective advantage for tumor cells during systemic treatments. Therefore, the next step would be to explore the predictive role of RCBTB1 expression on response to gemcitabine and docetaxel (GD) combination in the randomized study comparing gemcitabine alone (G) versus GD [17]. One can hypothesize that GD and G will be equivalent in sarcomas harboring *RCBTB1* deletion whereas GD will be more effective than G on sarcomas with preserved *RCBTB1* expression. Nevertheless, although we did not observe in vitro any sensitization effect on IB112 cells after gemcitabine or doxorubicin treatment in our experimental conditions, it is not excluded that high *RCBTB1* expression could sensitize other sarcoma models to other chemotherapies than docetaxel. Indeed, Zhou et al. (2010) reported that downregulation of *RCBTB1* in epithelial cells reduces cisplatin-induced apoptosis [15].

Unexpectedly, we also observed that higher expression of *RCBTB1* increases the proportion of mitotic LMS cells in vitro and promotes tumor growth in vivo. Reciprocally, *RCBTB1* downregulation dramatically decreased proliferation of dedifferentiated liposarcoma cells. These results are in conflict with the study of Zhou et al. (2010)*,* which reported a growth suppressive activity of *RCBTB1* in U2OS cells in vitro [15]. However, the impact of RCBTB1 on cell proliferation may be dependent on cellular context. Furthermore, its pro-proliferative action in our sarcoma cell lines provides at least one explanation for the efficacy of docetaxel, which targets mitotic cells, depending on the *RCBTB1* expression level.

The surprising pro-proliferative effect of RCBTB1 appears to be contradictory with our hypothesis of a tumor suppressor gene, coming out of our clinical data demonstrating that *RCBTB1* is frequently deleted and that its downregulation is prognostic for a higher risk for developing metastases. This assumption was also encouraged by the fact that *RCBTB1* is located in the 13q14 region [15,18], which comprises *RB1*, a well-known tumor suppressor and among the most frequently altered gene in those sarcomas [8]. The fact that *RCBTB1* and *RB1* have antagonistic roles in cell growth and are frequently co-deleted, as in 50% of our 106 sarcomas, suggests that *RCBTB1* may not be just a passenger gene affected by *RB1* deletion. Its inactivation in addition to *RB1* loss could avoid excessive cell proliferation, which could be deleterious for tumor development in terms of nutrient and energy expenditure for example but also in terms of response to treatment. Thus, co-deletion of *RCBTB1* and *RB1* might represent a selective advantage over cells only deleted for *RB1*. This underlines also how carefully results arising from clinical data must be interpreted about the role of a commonly altered gene. Indeed, we notice here that RCBTB1 rather acts as an oncogene at cellular level by promoting tumor growth but might physiologically favor a less invasive phenotype, thus clinically appearing as tumor suppressor gene.

The idea that *RB1* is not the only target of the frequent deletions in this chromosomal region is reinforced by the fact that in the cohort of 106 sarcomas, 7% of tumors are deleted only for *RB1*, whereas 13% carry a deletion that only affects *RCBTB1*. Furthermore, in contrast to *RCBTB1*, *RB1* is not associated with metastatic evolution in our cohort (Supplementary Figure S5). This does not challenge the major and indisputable role of *RB1* in pleomorphic sarcoma oncogenesis, but rather suggests that, in our cohort, the prognostic value of *RCBTB1* is independent of *RB1* status. Altogether, this rules out the hypothesis that *RCBTB1* is only a passenger gene affected by *RB1* deletion. Deletion of this and other chromosomal regions could indeed participate in a larger oncogenic program based on copy number variations that simultaneously affect multiple weak drivers to exert a cumulative effect equivalent to a single potent driver [19]. Furthermore, since we did not observe in vitro a direct effect of RCBTB1 on migratory capacities of sarcoma cells, this aspect should be examined in vivo, to understand if this negative result is due to the limitations of two-dimensional culture systems (absence of relevant microenvironment, no chemical or physical gradient, etc.) or if the link between RCBTB1 loss and metastasis mainly relies on adaptative capacities and selective advantage for cancer cells. *RCBTB1* deletion could possibly take part in the “Go or Grow” mechanism, which triggers a switch from a proliferative to an invasive phenotype in response to environmental stresses such as hypoxia, as demonstrated in other cancers [20,21]. This hypothesis offers new possibilities for investigation and could provide an additional explanation for the association between *RCBTB1* loss and metastatic progression.

Further investigation should identify the mechanisms and alterations that enable deleted cells (like IB112 and IB136) to continue the cell cycle, as *RCBTB1* downregulation dramatically decreases the proliferation of the LPS80 cell line. One research direction could be the regulation of sarcoma cell cycle by cullin3, which interacts with RCBTB1 for specific substrate degradation [22], as described in other cellular models [23]. Indeed, cullin3 recruits substrates for ubiquitination through polypeptide adaptors containing a BTB domain and these complexes have been reported as tumor suppressors since they regulate degradation of substrates involved in oxidative stress and cell cycle [24,25]. Consequently, RCBTB1 loss could result in accumulation of oncoproteins and in the dysregulation of cellular processes such as mitosis and microtubule dynamics, in turn leading to resistance towards anti-mitotic drugs. Understanding these mechanisms will probably shed light on a central role for *RCBTB1* deletion in tumor development, as suggested by our bioinformatic analysis and our in vivo experiments.

## 4. Materials and Methods

### 4.1. Ethics Statement

The samples used in this study are part of the Biological Resources Center of Institut Bergonié (CRB-IB, Bordeaux, France). In accordance with the French Public Health Code (articles L.1243-4 and R.1243-61), the CRB-IB has received the agreement from the French authorities to deliver samples for scientific research (number AC-2008-812). Expression and clinical data are extracted from ATGsarc database (http://atg-sarc.sarcomabcb.org/; restricted access) which integrate array data and clinical annotations from declared and approved French Sarcoma Group databases. The sarcoma tumor banks and databases received authorizations from the Advisory Committee on Information Processing in Material Research in the Field of Health (CCTIRS) and the French Data Protection Authority (CNIL). The CCTIRS approval was obtained on 24 November 2009; CNIL approval (No. 909510) was obtained on 5 February 2010. Written informed consent was received from participants before inclusion in the study. Every case was histologically reviewed by the pathologist subgroup of the French Sarcoma Group and classified according to the 2013 World Health Organization classification by histology, immunohistochemistry, and molecular genetics when needed.

### 4.2. Sample Description

The first cohort (#1) is a training set of 106 sarcomas with complex genomics, with genomic and transcriptomic data. The second cohort (#2) is a validation set of 204 sarcomas analyzed by gene expression microarray. Cohorts #1 and #2 are described in Table 1. The last cohort (#3, SRP057793) is a set of 145 sarcomas with RNA-seq expression, as previously described [11]. Among the 145 tumors, 14 tumors were metastatic at the time of diagnosis.

### 4.3. Chemicals

Doxorubicin (Adriamycin; Pfizer, New York, NY, USA) and gemcitabine (Gemzar; Eli Lilly and Company, Neuilly-sur-Seine, France) were obtained from the pharmacy of the Institut Bergonié (Bordeaux, France). Docetaxel (Taxotere; Sanofi-Aventis, Gentilly, France) was obtained from Sigma Aldrich (#01885, St. Quentin Fallavier, France).

### 4.4. Cell Lines

Sarcoma cell lines were established as previously described [25]. Authentication of cell line was performed by array CGH and compared with the corresponding original tumor. Sarcoma cell lines IB112, IB136, and LPS80 were cultured in RPMI-1640/GlutaMAX-I (Life Technologies Inc., brand of ThermoFisher Scientific, Waltham, MA, USA) supplemented with 10% Foetal Bovine Serum (FBS) and 1% penicillin–streptomycin (Life Technologies Inc.). The HEK-293T cell line was cultured in DMEM/GlutaMAX-I (Life Technologies Inc.) supplemented with 10% FBS and 1% penicillin–streptomycin (Life Technologies Inc.). Cells were grown at 37 °C in a humidified atmosphere containing 5% CO_2_.

IB112 and IB136 were infected with a lentiviral vector containing the cDNA sequence of RCBTB1 coupled to the HA-tag under the control of a CMV promoter (EX-Z7772-Lv120, GeneCopoeia, Rockville, MD, USA). Control cell line was established with lentiviral transduction of an empty vector (pReceiver-Lv105, GeneCopoeia).

LPS80 was infected with a lentiviral vector containing a shRNA sequence under the control of Tet promoter. The control cell line expresses a shRNA targeting no human RNA (“sh Non-Targeting”).

Sequences of shRNA targeting RCBTB1: #1:CCGGGCTTATGTGGAAAGAAGATTACTCGAGTAATCTTCTTTCCACATAAGCTTTTTTG; #2: CCGGCTGGACAATGGCGAGGTATATCTCGAGATATACCTCGCCATTGTCCAGTTTTTTG.

Expression of shRNA is induced by doxycycline (2 µg/mL, #D9891, Sigma Aldrich, St. Quentin Fallavier, France).

For lentiviral transduction, Vesicular Stomatitis Virus Glycoprotein (VSV-G)-pseudotyped lentiviral particles were produced by co-transfection of 293T cells with previous vectors and the compatible packaging plasmids psPAX2 and pVSVg. Cell lines were incubated overnight with lentiviral supernatants in presence of polybrene (8 μg/mL, #H9268, Sigma Aldrich, St. Quentin Fallavier, France). Stably transduced cells were selected with addition of puromycin (2 µg/mL, #P9620, Sigma Aldrich, St.) into culture medium.

### 4.5. Comparative Genomic Hybridization (aCGH)

Genomic DNA was extracted using the standard phenol-chloroform extraction protocol [7]. Affymetrix SNP array 6.0 (Affymetrix, brand of ThermoFisher Scientific, Waltham, MA, USA) was used according to the manufacturer’s instructions. Normalization of 106 samples and 5 normal control DNAs was performed with the Genotyping console 2.0 software (Affymetrix).

### 4.6. Gene Expression Microarray

Total RNAs were extracted as described previously [8]. RNA quality was checked on an Agilent 2100 Bioanalyzer (Agilent Technologies, Santa Clara, CA, USA). Samples were then analyzed by Human Genome U133 Plus 2.0 array (Affymetrix), according to the manufacturer’s procedures.

### 4.7. Bioinformatics Analyses Pipeline for the Identification of Driver Genes

Data were analyzed with home-made Perl (v 5.10) and R scripts (2.14) and with the bioconductor package “GO.db”.

On the genomic array, probes meeting the following criteria were excluded: probes present in referenced constitutional copy number variation (CNV) regions, probes targeting sexual chromosomes, probes that are not localized in a gene and probes that are altered in control samples. In all, 512,055 probes were analyzed, representing 12,124 genes. A unique status was assigned to each gene based on the information gathered by all probes: normal, lost or gained. Among the 12,124 genes studied at the genomic level, 11,447 genes were present on the Affymetrix U133 Plus 2.0 array. For each gene, one probe was selected as representative of the expression of the gene, based on the maximum interquartile range (IQRmax) of all probes targeting the given gene. For further analysis, we considered that a gene was altered when its genomic status is lost or gained and when its expression is under the first quartile or above the third quartile of control group expression, which is calculated on at least 30 samples for which the genomic status of a given gene is normal.

Among the 11,447 genes studied at the transcriptomic level, 8527 genes were listed in the Gene Ontology (GO) at the time of the analysis. We considered that a GO biological pathway was involved in oncogenesis when at least one gene of the pathway was altered. The enrichment of a biological pathway or of an altered gene in a subgroup was tested with a Fisher’s exact test. A gene was considered as a candidate “driver” gene if both its biological pathway and its alteration were enriched in a subgroup.

### 4.8. RNA Sequencing

The process from RNA extraction to final BAM files was previously described [11]. We used SAMtools and BCFtools (v0.1.19) [26,27] with custom depths (at least two alternate bases and five total bases) for reporting a candidate variant. These variants were then annotated by ANNOVAR (October 2013) [28] with hg19 genome version, transcriptome annotations (November 2013) and following databases of observed variants: dbSNP (v138) [29], 1000 G project (April 2012) [30], ClinVar (September 2014) [31], and COSMIC (v70) [32].

### 4.9. Taqman Assay

cDNAs were synthesized from 1 μg of RNA using the GeneAmp RNA PCR core Kit (Applied Biosystems, brand of ThermoFisher Scientific, Waltham, MA, USA). Quantitative PCR analyses were performed using TaqMan Assays-on-demand Gene expression reagents (Applied Biosystems) with qPCR Mastermix Plus without UNG (Eurogentec, Seraing, Belgium). We used the TaqMan Gene Expression assays provided by Applied Biosystems. The assay IDs were as follows: Hs00216991_m1 for *RCBTB1* and Hs99999902_m1 for RPLP0. For results normalization, *RPLP0* was used as a reference gene.

### 4.10. Western Blot

Cells were rinsed with ice-cold PBS and lysed for 30 min at 4 °C in RIPA lysis and extraction buffer (#R0278, Sigma Aldrich, St.) supplemented with a protease/phosphatase inhibitor cocktail (#11697498001, Roche, Basel, Switzerland). Lysates were pelleted for 10 min at 13,000× *g* at 4 °C and supernatants were collected for protein quantitation (DC protein assay kit, Biorad, Hercules, CA, USA). After denaturation, 40 µg total proteins of each sample were separated by sodium dodecyl sulfate–polyacrylamide gel electrophoresis, and transferred to polyvinylidene difluoride membranes (iBlot2, ThermoFisher Scientific, Waltham, MA, USA) for membrane blocking and immunoblotting with the primary antibody (anti-HA, #sc-805, Santa Cruz Biotechnology, Dallas, TX, USA) at 4 °C overnight. After washing, blots were incubated for 1 h with a horseradish peroxidase-linked anti-rabbit antibody (Amersham, brand of GE Healthcare Europe GmbH, Velizy-Villacoublay, France) and processed for chemiluminescent substrate (Amersham ECL Select detection reagent kit, Sigma Aldrich, St.) according to the manufacturer’s instructions. Signal was detected using Fusion Fx7 (Thermo Fisher Scientific, Waltham, MA, USA) imaging system. β-actin (#A5316, Sigma Aldrich, St.) was used as a loading control.

### 4.11. Apoptosis Assay

To perform the assay, 75,000 cells were seeded into 12-well plates in three replicates. The day after, culture medium was replaced by drug-containing medium. After 72 h of incubation, cell death was measured by dual Annexin V-FITC/PI staining (#556547, BD Biosciences, San Jose, CA, USA) according to manufacturer’s recommendations. For doxorubicin, whose red coloration impairs PI staining, we analyzed only AnnexinV-FITC staining.

For each sample, 10,000 cells were analyzed by flow cytometry (FACS Calibur, BD Biosciences). Data were acquired using BD CellQuestPro software. Data analysis was performed with FlowJo v10.1 (FlowJo LLC, Ashland, OR, USA) and Prism6 v6.01 (GraphPad Software Inc., La Jolla, CA, USA) software.

### 4.12. In Vivo Experiments

NSG (NOD scid gamma, strain NOD.Cg-Prkdcscid Il2rgtm1Wjl/SzJ) mice received on the right flank a subcutaneous injection of 2.5 × 10^6^ cells. Tumor size was measured twice a week using a caliper and calculated using the formula: V = length × width^2^/2. When tumors reached a volume of 150 mm^3^, mice were treated with two intraperitoneal injections spaced by 4 days of DMSO or docetaxel at 1 mg/mL or docetaxel at 2 mg/mL. Twenty-eight days after the first treatment, mice were sacrificed. Tumors were recovered and weighed.

Animals were maintained under specific pathogen-free conditions in the animal facility of Bordeaux University (Bordeaux, France). Experiments were performed in conformity with the rules of the Institutional Animal Care and Use committee (approval number DIR13109) and all efforts were made to minimize animal suffering.

### 4.13. Immunofluorescence

Cells were seeded into 96-well imaging plates (#CLS3603, Sigma), incubated with drugs as indicated and fixed with 3.7% formaldehyde for 20 min at room temperature (RT). Then, cells were washed three times with PBS and permeabilized in 0.5% Triton X-100 for 5 min. Nonspecific binding was avoided by blocking with 0.2% gelatin/PBS for 30 min. Nuclei were stained with Hoechst 33,258 dye (Molecular Probes, brand of ThermoFisher Scientific, Waltham, MA, USA).

Staining was performed using a specific primary anti-HA (#sc-805 Santa Cruz Biotechnology, Dallas, TX, USA) antibody and anti-pMPM2 antibody (#05-368, Merck Millipore, brand of Merck KGaA, Darmstadt, Germany) overnight at 4 °C and a fluorescent secondary antibody conjugate (Alexa Fluor 488, anti-mouse and Alexa Fluor 594, anti-rabbit, Molecular Probes, brand of ThermoFisher Scientific, Waltham, MA, USA) for 1 h at RT. Cells were analyzed using a Leica DMi8 epifluorescent microscope (Leica Microsystems, Wetzlar, Germany) with appropriate filters. Pictures were captured using a Hamamatsu C11440-CCD camera and the Leica Application Suite X software (Leica Microsystems). To generate these results, three independent experiments with 15 replicates have been performed. For each replicate, four different fields were acquired. In this way, an average of 100 cells was analyzed for each replicate. The detection threshold for RCBTB1-HA-positive cells was set according to empty-vector expressing cells. For p-MPM2-positive cells, intense-green cells with mitotic figures (observed with Hoechst staining) were identified in control conditions, and the same detection threshold was applied to all conditions.

### 4.14. Proliferation Assays

In 96-well plates, 1000 cells were seeded in five replicates. Every 2 to 3 days, cells were washed, trypsinized, and harvested in a final volume of 200 µL PBS. The number of viable cells was evaluated by flow cytometry (FACS Calibur, BD Biosciences) based on their morphological features. Culture medium was changed at days 1, 3 and 6 with or without addition of doxycycline (2 µg/mL). Data were acquired using BD CellQuestPro software and analyzed using FlowJo (FlowJo LLC) and Prism6 v6.01 (GraphPad Software Inc., La Jolla, CA, USA) software.

### 4.15. Statistical Analysis

Metastasis-free survival (MFS) was defined as the interval between diagnosis and the time of distant recurrence or the last follow-up. Survival rates were estimated using the Kaplan-Meier method and compared using the log-rank test and hazard ratios (HR). Descriptive statistics were used to show the distribution of variables in the population. All statistical tests were two-sided, and *p* < 0.05 indicated statistical significance. All statistical analyses were carried out using the R statistical environment (v 3.3.2).

Each experiment was repeated at least three times. For examining the statistical significance of the results, analyses were performed with Prism6 v 6.01 (GraphPad Software Inc.) software. Normal distribution of data sets was examined with a Shapiro–Wilk normality test. If data passed normality test, statistical significance between two or more conditions was assessed with an unpaired *t*-test or an ANOVA (Holm–Sidak’s multiple comparisons test), respectively, and results were represented as mean ± SD. Otherwise, a Mann–Whitney test (for two groups) or a Kruskal–Wallis test (Dunn’s multiple comparisons test, to compare more than two groups) was used and medians with interquartile range (IQR) were plotted. Significant differences are represented as * if *p*-value *p* < 0.05, ** if *p* < 0.01 and *** if *p* < 0.001 on all figures.

## 5. Conclusions

In conclusion, this study shows that RCBTB1 is commonly deleted in sarcomas with complex genetics and is associated with metastatic progression. Yet, there is a lack of knowledge about the physiologic role of RCBTB1 and our work provides novel evidence about the impact of RCBTB1 on cell cycle and drug resistance both in vitro and in vivo. This deletion might belong to a larger oncogenic program triggering a fine tuning of cell proliferation, thus allowing tumor progression and promoting drug resistance.

## Figures and Tables

**Table 1:** Patient characteristics of cohorts #1 and #2. UPSs: undifferentiated pleomorphic sarcomas.

Clinical Characteristics	Cohort #1 (106 Sarcomas)	Cohort #2 (204 Sarcomas)
Median age at diagnosis (95% CI)	63 (59–66)	63 (61–67)
Median follow-up in months (95% CI)	20.40 (13.80–27.07)	29.04 (22.68–35.28)
Histotypes (%)		
Leiomyosarcomas	33 (31.13)	60 (29.41)
Undifferentiated Pleomorphic Sarcomas	38 (35.85)	82 (40.20)
Myxofibrosarcoma	23 (21.70)	0
Pleomorphic liposarcomas	7 (6.60)	0
Dedifferentiated liposarcomas	0	62 (30.39)
Others	5 (4.72)	0
Gender (%)		
Male	55 (51.89)	103 (50.49)
Female	51 (48.11)	101 (49.51)
Metastasis (%)	32 (30.19)	83 (40.69)
Local recurrence (%)	23 (21.70)	49 (24.02)
FNCLCC Grades (%)		
Grade 1	8 (7.55)	12 (5.88)
Grade 2	25 (23.58)	69 (33.82)
Grade 3	68 (64.15)	103 (50.49)
Unknown	5 (4.72)	20 (9.80)
Tumor size (cm) (95% CI)	9 (8–10)	10 (10–11)
Location (%)		
External Trunk	88 (83.02)	151 (74.02)
Trunk wall	25 (23.59)	31 (15.20)
Head and neck	1 (0.94)	2 (0.98)
Extremities	62 (58.49)	118 (57.84)
Internal Trunk	15 (14.15)	53 (25.98)
Unknown	3 (2.83)	0 (0)
Stage at Diagnosis (%)		
Localized	100 (94.3)	188 (92.2)
Metastatic	6 (5.7)	16 (7.8)

## Supplementary Materials

The following are available online at http://www.mdpi.com/2072-6694/11/1/81/s1. Table S1: List of the 41 biological pathways referenced in Gene Ontology database commonly altered in 100% of sarcomas, Table S2: Description of the repartition of the 162 tumor samples according to histotype and RCBTB1 genomic status, Table S3: Repartition of cases of metastatic disease according to RCBTB1 expression level in tumors (high or low, cut-off = median), Figure S1: Boxplot of RCBTB1 expression (RNA Seq V2 RSEM) in the TCGA cohort of 162 soft tissue sarcomas, according to RCBTB1 genomic status, Figure S2: Apoptotic rate of IB112 cells expressing an empty vector or RCBTB1-HA after 72 h incubation with doxorubicin or gemcitabine, Figure S3: Cell migration of IB112 cell line expressing RCBTB1, Figure S4: Follow-up of tumor volume during treatment. Figure S5: Metastasis-free survival analysis of sarcoma patients in cohort #1 according to RB1 genomic status or expression level.
