# Supplementary material for: RCBTB1 Deletion Is Associated with Metastatic Outcome and Contributes to Docetaxel Resistance in Nontranslocation-Related Pleomorphic Sarcomas
Source: Cancers (Basel). 2019 Jan 11;11(1):81. doi: 10.3390/cancers11010081 (PMC6356223; doi:10.3390/cancers11010081)
Supplement: Supplementary file 1 [file cancers-11-00081-s001.pdf]

Supplementary Materials: RCBTB1 deletion is associated with metastatic outcome and contributes to docetaxel-resistance in non-translocation related pleomorphic sarcomas

Mauduit Olivier, Brulard Céline, Lesluyes Tom, Delcroix Vanessa, Pérot Gaëlle, Choublier Nina, Michaud Mickael, Baud Jessica, Lagarde Pauline, Aurias Alain, Coindre Jean-Michel, Lartigue Lydia, Blay Jean-Yves and Chibon Frédéric

Table S1. List of the 41 biological pathways referenced in Gene Ontology database commonly altered in 100% of sarcomas.

ID	Biological Pathways of GO Database:	*p*-Value
GO:0006996	organelle organization	1.26e-88
GO:0007049	cell cycle	8.87e-81
GO:0016070	RNA metabolic process	3.52e-80
GO:0044267	cellular protein metabolic process	6.96e-78
GO:0007399	nervous system development	1.21e-77
GO:0090304	nucleic acid metabolic process	2.05e-77
GO:0030154	cell differentiation	1.29e-76
GO:0006950	response to stress	1.50e-76
GO:0048869	cellular developmental process	1.55e-76
GO:0010467	gene expression	3.13e-76
GO:0048731	system development	7.40e-75
GO:0006139	nucleobase, nucleoside, nucleotide and nucleic acid metabolic process	5.63e-73
GO:0022008	neurogenesis	1.75e-72
GO:0009605	response to external stimulus	2.09e-72
GO:0007275	multicellular organismal development	6.04e-72
GO:0019538	protein metabolic process	8.07e-72
GO:0048699	generation of neurons	8.60e-72
GO:0048856	anatomical structure development	1.20e-70
GO:0071842	cellular component organization at cellular level	3.35e-70
GO:0016043	cellular component organization	3.35e-70
GO:0030182	neuron differentiation	7.44e-70
GO:0071841	cellular component organization or biogenesis at cellular level	1.31e-69
GO:0071840	cellular component organization or biogenesis	1.31e-69
GO:0042221	response to chemical stimulus	2.71e-69
GO:0034641	cellular nitrogen compound metabolic process	4.16e-66
GO:0032502	developmental process	1.18e-65
GO:0006807	nitrogen compound metabolic process	4.30e-63
GO:0007165	signal transduction	1.13e-60
GO:0023052	signaling	2.52e-57
GO:0051716	cellular response to stimulus	2.00e-52
GO:0032501	multicellular organismal process	6.63e-51
GO:0044260	cellular macromolecule metabolic process	1.92e-47
GO:0050896	response to stimulus	7.55e-41
GO:0043170	macromolecule metabolic process	2.50e-40
GO:0044237	cellular metabolic process	1.79e-30
GO:0044238	primary metabolic process	2.15e-29
GO:0050794	regulation of cellular process	2.97e-27
GO:0050789	regulation of biological process	4.02e-23
GO:0065007	biological regulation	1.43e-19
GO:0008152	metabolic process	1.32e-17
GO:0009987	cellular process	0.04

Table S2. Description of the repartition of the 162 tumor samples according to histotype and RCBTB1 genomic status.

**Tumor type (*N* = 162)**	**Homozygous Deletion**	**Heterozygous Deletion**	**Normal**	**Gain**
Dedifferentiated Liposarcoma (*N* = 22)	0	9 (40.9%)	13 (59.1%)	0
Leiomyosarcoma (*N* = 60)	11 (18.3%)	41 (68.3%)	7 (11.7%)	1 (1.7%)
Malignant Peripheral Nerve Sheath Tumor (*N* = 7)	0	5 (71.4%)	2 (28.6%)	0
Myxofibrosarcoma (*N* = 19)	1 (5.3%)	14 (73.7%)	4 (21.1%)	0
Pleomorphic Liposarcoma (*N* = 2)	0	0	2 (100%)	0
Synovial Sarcoma (*N* = 9)	0	1 (11.1%)	8 (88.9%)	0
Undifferentiated Pleomorphic Sarcoma/Malignant Fibrous Histiocytoma/High-Grade Spindle Cell Sarcoma (*N* = 42)	8 (19%)	28 (66.7%)	5 (11.9%)	1 (2.4%)
Soft Tissue Sarcoma (*N* = 1)	1 (100%)	0	0	0
total (%)	13.0	60.5	25.3	1.2

Table S3. Repartition of cases of metastatic disease according to *RCBTB1* expression level in tumors (high or low, cut-off = median).

**Metastatic Disease?**	No	Yes
low *RCBTB1* expression	*n* = 48 (59%)	*n* = 33 (41%)
high *RCBTB1* expression	*n* = 60 (74%)	*n* = 21 (26%)

Chi-squared test, p=0.046.

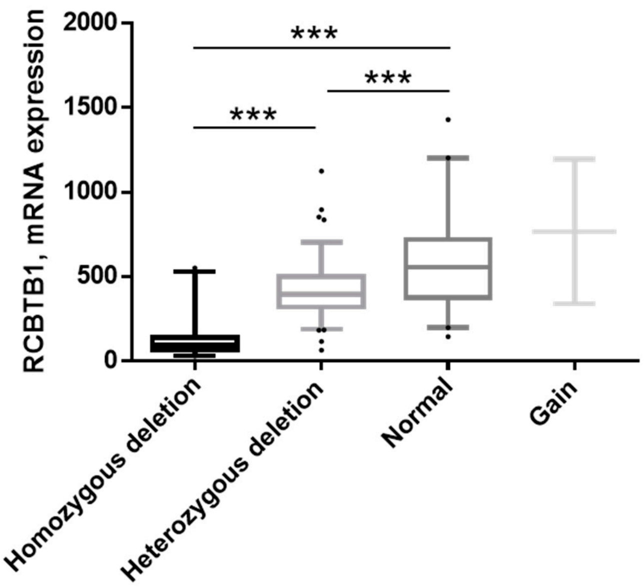

Figure S1. Boxplot of *RCBTB1* expression (RNA Seq V2 RSEM) in the TCGA cohort of 162 soft tissue sarcomas, according to *RCBTB1* genomic status. Line in the middle of the box is the median, whiskers are drawn down to the 5th percentile and up to the 95th. Points below and above the whiskers are represented as individual points. ***: $p < 0.001$.

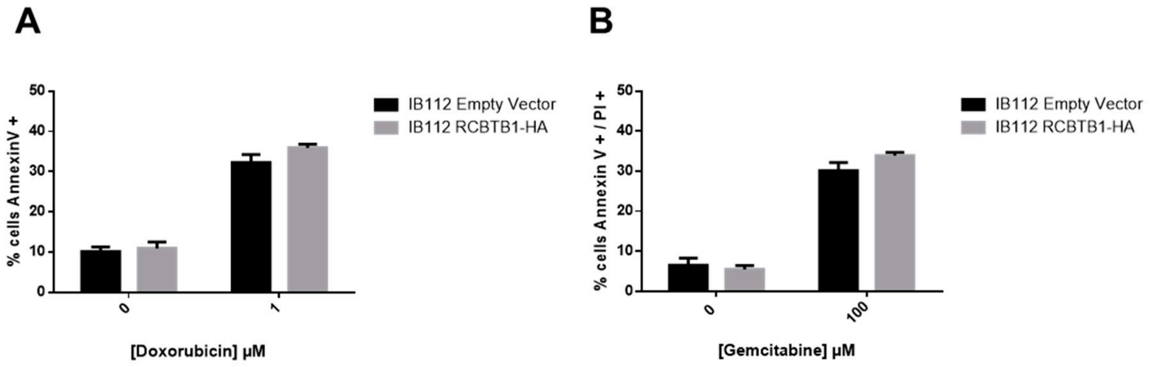

Figure S2. Apoptotic rate of IB112 cells expressing an empty vector or RCBTB1-HA after 72 h incubation with (**A**) doxorubicin or (**B**) gemcitabine. Apoptosis induced by (**A**) doxorubicin or (**B**) gemcitabine was measured by dual Annexin V-FITC / PI staining (except for doxorubicin, for which simple Annexin V-FITC staining was used). 10,000 cells were analyzed by flow cytometry for each sample. Histograms sum up three experiments with three replicates. Data are represented as mean (SD).

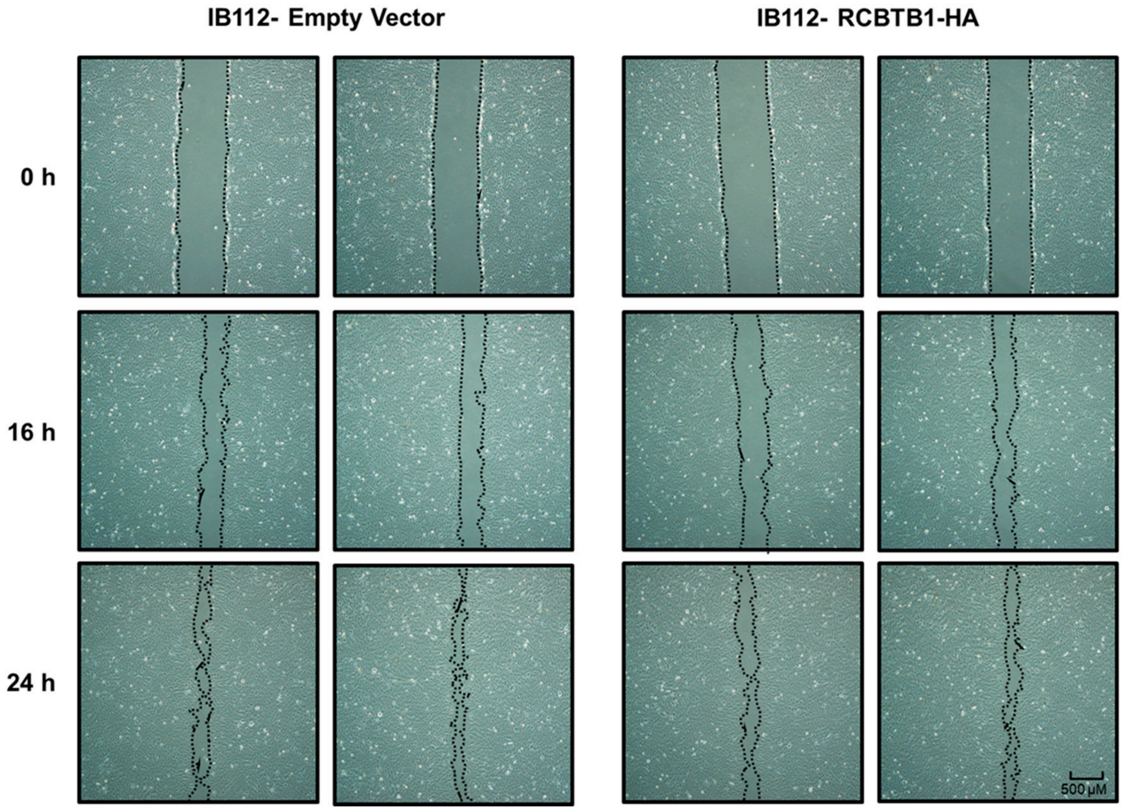

Figure S3. Cell migration of IB112 cell line expressing RCBTB1. RCBTB1 was overexpressed by lentiviral transduction in the IB112 LMS cell line. Cell migration was assessed by wound-healing assays. When cells reached confluence, cell monolayer was scratched with a pipette tip and culture medium was changed. Photos were taken at 0, 16 and 24 h after the wound was performed. Data shown are duplicates representative of the results obtained in three independent experiments.

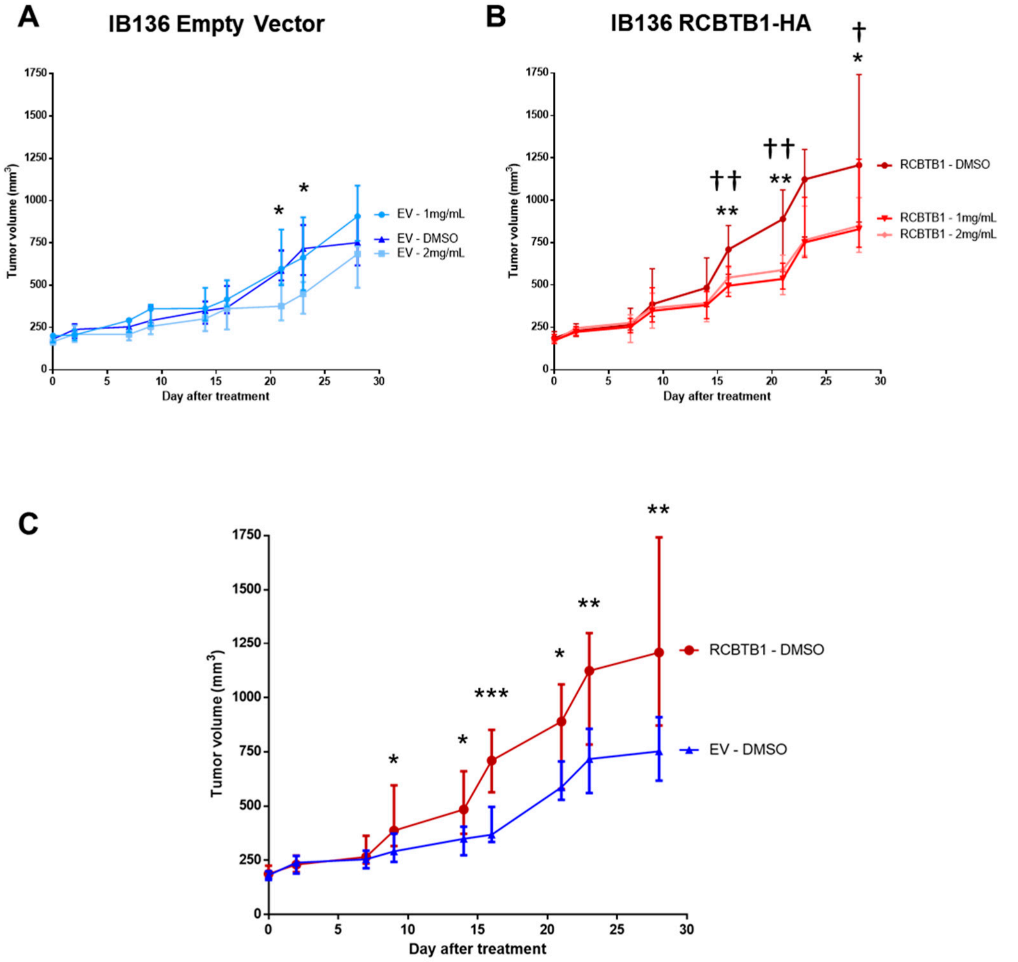

Figure S4. Follow-up of tumor volume during treatment. (**A**–**B**) Evolution of tumor volume from the beginning of treatment until sacrifice in groups of mice engrafted with (**A**) IB136 Empty vector cells or (**B**) IB136 RCBTB1-HA cells. † or * indicate statistical significance (p < 0.05) between the group treated with DMSO and the one treated with 1 mg/mL or 2mg/mL docetaxel, respectively. (**C**) Comparison of tumor growth during treatment between each control group treated with DMSO. **(A–C)** Data are represented as median (IQR). † or * indicate p < 0.05, ** or †† mean p < 0.01 and *** is for p < 0.001.

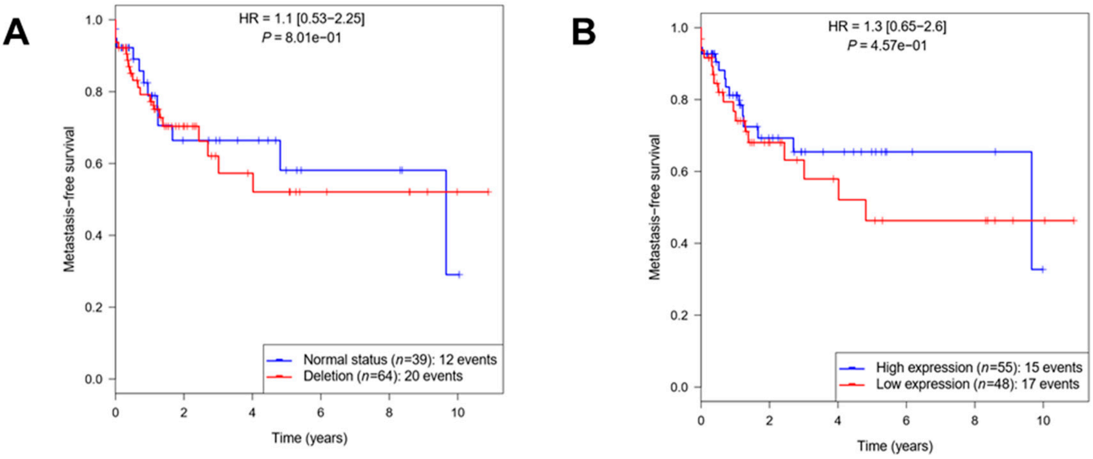

Figure S5. Metastasis-free survival analysis of sarcoma patients in cohort #1 according to *RB1* genomic status (**A**) or expression level (**B**).

© 2019 by the authors. Submitted for possible open access publication under the terms and conditions of the Creative Commons Attribution (CC BY) license (http://creativecommons.org/licenses/by/4.0/).
